# Association between dietary fat intake and colorectal cancer: A multicenter case-control study in Iran

**DOI:** 10.3389/fnut.2022.1017720

**Published:** 2022-11-16

**Authors:** Monireh Sadat Seyyedsalehi, Giulia Collatuzzo, Inge Huybrechts, Maryam Hadji, Hamideh Rashidian, Roya Safari-Faramani, Reza Alizadeh-Navaei, Farin Kamangar, Arash Etemadi, Eero Pukkala, Marc J. Gunter, Veronique Chajes, Paolo Boffetta, Kazem Zendehdel

**Affiliations:** ^1^Department of Medical and Surgical Sciences, University of Bologna, Bologna, Italy; ^2^Cancer Research Center, Cancer Institute, Tehran University of Medical Sciences, Tehran, Iran; ^3^International Agency for Research on Cancer, Lyon, France; ^4^Health Sciences Unit, Faculty of Social Sciences, Tampere University, Tampere, Finland; ^5^Research Center for Environmental Determinants of Health, School of Public Health, Kermanshah Medical Sciences University, Kermanshah, Iran; ^6^Gastrointestinal Cancer Research Center, Non-communicable Diseases Institute, Mazandaran University of Medical Sciences, Sari, Iran; ^7^Department of Biology, School of Computer, Mathematical, and Natural Sciences, Morgan State University, Baltimore, MD, United States; ^8^Digestive Oncology Research Center, Digestive Diseases Research Institute, Shariati Hospital, Tehran University of Medical Sciences, Tehran, Iran; ^9^Metabolic Epidemiology Branch, Division of Cancer Epidemiology and Genetics, National Cancer Institute, Bethesda, MD, United States; ^10^Finnish Cancer Registry - Institute for Statistical and Epidemiological Cancer Research, Helsinki, Finland; ^11^Stony Brook Cancer Center, Stony Brook University, Stony Brook, NY, United States; ^12^Cancer Biology Research Center, Cancer Institute, Tehran University of Medical Sciences, Tehran, Iran

**Keywords:** gastrointestinal neoplasms, food frequency questionnaire, fat, diet, colorectal cancer

## Abstract

The evolving trends in colorectal cancer (CRC) as one of the most common malignancies worldwide, have likely been influenced by the implementation of screening programs and changes in lifestyle habits. Changing lifestyle, including the shift in diet composition with higher fat, sugar, and animal-source foods intake, led to an increasing burden of CRC in countries undergoing rapid socioeconomic improvement. Results for the link between specific fatty acids (FAs) and CRC are generally inconclusive and more limited in developing countries than elsewhere. This study aims to investigate the association between FA intakes and CRC and its anatomical subsites in a large Iranian case-control study. A food frequency questionnaire was used to collect information on dietary intake in 865 cases and 3206 controls. We conducted multivariate logistic regression models to calculate the odds ratio (OR) and 95% confidence interval (CI). We found positive association between CRC and high intake of dietary total fat (OR highest quartile _Q4_ = 1.77, 95% CI = 1.32–2.38), cholesterol (OR_Q4_ = 1.58, 95% CI = 1.22–2.05), and palmitoleic acid (OR_Q4_ = 2.16, 95% CI = 1.19, 3.91), and an inverse association with high intake of dietary heptanoic acid (OR_Q4_ = 0.33, 95% CI = 0.14, 0.79) and low intake of palmitic acid (OR lowest quartile _Q2_ = 0.53, 95% CI = 0.31–0.88). None of the fat variables were associated with rectal cancer. Our study suggests that the recommendation of limited consumption of fats may decrease the risk of CRC among the Iranian population.

## Introduction

Colorectal cancer (CRC) is the third most common malignancy and the second leading cause of cancer death, with an estimated 1.8 million new cases and 900,000 deaths in 2020 worldwide (1). A higher incidence rate is found in men and in highly industrialized countries (2). In Iran, CRC is the fourth most common cancer, with an age-adjusted incidence rate (ASR) of 15.9 and 11.9 for men and women, respectively (1). From 2016 to 2020, previous studies identified an increasing trend in Iran and predicted a 54% increase by 2025 (3). The changing trends in CRC have likely been influenced by two factors: (i) the implementation of screening programs; (ii) changes in lifestyle habits. Colonoscopy as a gold standard for CRC screening has on the one hand the short-term effect of apparent increasing CRC incidence due to early detection; on the other, it has been proven to reduce the incidence in the long term by the removal of precancerous lesions (4). The changing lifestyle, including the shift of diet composition toward a “westernized” pattern connoted by higher fat, sugar, and animal-source foods intake, leads to an increasing burden of CRC in countries undergoing rapid socioeconomic improvement. These situations can lead to a growing concern regarding the rising number of CRC cases, in particular among those <50 years of age (4, 5). In particular, CRC has observed an increase in the Iranian population, especially among men and in more urbanized areas (6, 7). The investigation of dietary behaviors in Iranians have been involved different study groups, being described for example by Saneei et al. Different dietary patterns have been described by residency in Iran, with higher intake of vegetables, fruit, meat, fat, saturated fatty acids (SFA), cholesterol, vitamin C, and beta-carotene and less bread, cereal, and carbohydrates in urban compared to rural dwellers. Also, similar differences in fat, SFA, MUFA, and bread consumption was seen by race, with Turkmens having higher intakes than non-Turkmens. These findings were related to the higher incidence of esophageal cancer registered in Iranians from low socioeconomic status and living in rural areas. Indeed, nutrition deficiencies may contribute to cancer by altering metabolism of carcinogens or by impairing DNA repair (8, 9). Zamaninour et al. had recently described the prevalence of unhealthy dietary habits in Iranian population, where more than 80% of people reported suboptimal intakes of fruit, dairy products and fish, and about 60% also reported suboptimal vegetables intake (10). The identification of dietary risk factor of CRC in Iranian population has been the aim of different recent studies. A case-control study conducted in Iran showed the correlation between a pro-inflammatory diet, based on high consumption of red and processed meat and fat, was associated to CRC and colorectal adenomatous polyps (11).

At least 12% of CRC cases are directly attributable to overweight/obesity according to a recent global review (12). Diets rich in fat are also a risk factor for obesity and cancer (13). According to the literature, different roles are exerted by different types of dietary fats depending on their source such as, ω-3 polyunsaturated fatty acids (PUFAs) play a role in protecting against adipose tissue inflammation, in contrast to omega-6 (ω-6) PUFAs and some saturated fatty acids (SFAs) and monounsaturated fatty acids (MUFAs), which promote inflammation (14, 15). A higher risk of CRC has been associated with high animal fat intake, but not vegetable fat (16). Although, the amount and type of different fatty acids (FAs) consumed by CRC patients resulted not to be associated either with recurrence or survival (17). To date, limited data provide the quantification of the effect exerted by FAs on CRC. Besides this, few results are available on the role played by the different types of FAs on CRC overall and by anatomical sub-sites.

Currently, screening programs in Iran are based on the identification of high-risk individuals, corresponding to first-degree relatives of CRC patients. Thus, they are based on the consultation of cancer registry. The first-degree families of CRC patients are invited to cancer screening and participate in counseling sessions, possibly leading to colonoscopy recommendation. So far, Iran does not have any screenings or plans for screening the general population at medium-risk of cancer. This makes Iran very different from most of the other countries, where non-invasive testing is purposed based on risk profile but also older age and are followed by the invasive endoscopic examination. This issue was addressed by Nikbakht et al. who conducted a study to investigate the use of immunochemical fecal occult blood test (IFOBT) in a mid-risk for CRC Iranian population. The study showed high responsiveness rates from the population, and high rates of positive IFOBT were found (18). Considering these evidence, better comprehension of CRC epidemiology in Iran results highly important for CRC control. In particular, the identification of risk factors of CRC may help preventing the disease and target high-risk population with secondary prevention, including colonoscopy.

This study aims to look at associations between fatty acid intakes and CRC risk considering overall and sub-site-specific CRC in a large Iranian case-control study. Our analysis provides useful data to deepen the knowledge on the role of fatty acids in CRC development. Moreover, we give valuable information on the consumption of fatty products in Iran and their relationship with CRC from different anatomical sites.

## Materials and methods

### Study design and population

**Figure 1:**
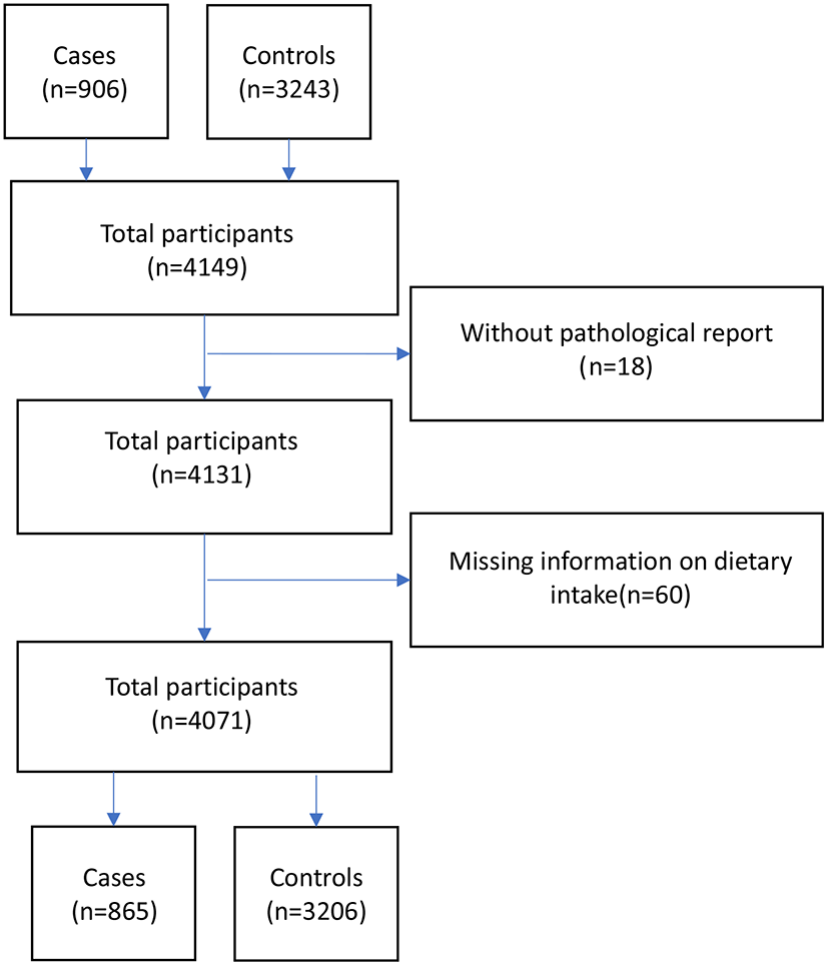
Flow diagram of the population enrollment based on the inclusion and exclusion criteria in IROPICAN study.

A total of 4,149 participants, including 906 CRC cases with pathologic diagnosis and 3,243 controls were recruited between May 2017 and 2020 from the main cancer clinic and hosptals in 7 provinces of Iran (Tehran, Fars, Mazandaran, Kerman, Golestan, Kermanshah, and Mashhad). They were part of the IROPICAN study, a multicenter case-control study coducted in 10 Iranian provinces, which was designed to examine the link between opium use and the development of lung, colorectal, bladder, and head and neck cancers (19). For the analysis of dietary intakes of fat, we excluded participants without the pathological report diagnoses confirmation (*n* = 18); those with missing information on dietary intake and those in the highest or lowest 1% of the distribution for the ratio of energy intake to estimated energy requirement (*n* = 60). We included 865 cases and 3,206 controls in our analysis (Figure 1). Controls were enrolled concurrently with the cases among the healthy visitors of non-oncology wards. The controls had to be free of cancer at the date of recruitment. The mean age at recruitment was 58.5 years and 57.1 years for the cases and controls, respectively. In this study, we estimated around 800 colorectal cancer cases using the OR and CI95 reported by previous studies (20–22), assuming 20 and 30% exposure prevalence among controls and 80% power.

### Ascertainment of CRC cases

CRC cases was defined by the International Classification of Diseases (ICD-O-3) as tumors of the colon (C18) or rectum (C19–C20). Colon cancer may be categorized as proximal (from cecum to splenic flexure, C18.0–C18.5) and distal (from descending colon to sigmoid colon, C18.6–C18.7), while rectum cancer occurs from the recto-sigmoid junction (C19) down to the rectum. Anus tumors were excluded from the study. Also, all histological types of CRC except melanoma and sarcoma were included.

### Anthropometry and lifestyle collection

During a face-to-face interview, lifestyle questionnaires were used to collect information about education, tobacco use, opium use, socioeconomic status (SES), physical activity, previous illness, and use of nonsteroidal anti-inflammatory drugs (NSAIDs). At the time of enrollment, trained professionals generally measured the standing height of participants (cm). The body mass index (BMI) was calculated as weight/height squared (kg/m2). Cases were asked about their body weight before cancer diagnosis, while controls were measured their body weight at the time of the interview. Based on the Finland Job Exposure Matrix (FINJEM) (23, 24) we analyzed the estimated perceived physical activity workload (PPWL). A principal component analysis was used to calculate the socioeconomic status (SES) of the participants, and the SES was calculated based on the number of years of education the participants had and whether they owned any assets like vacuum cleaners, clothes washers, dishwashers, freezers, internet access, microwaves, laptops, mobile phones, cars, and shops (25).

### Diet and fatty acids considered in the analysis

Dietary intakes were assessed with validated qualitatively Persian Cohort FFQ (26) administered by trained interviewers. The usual intake of 131 food items in separate parts including bread and cereal, meat, vegetables and fruits, dietary products, oils, sugars, species, and other group (113 items), dietary supplements (17 items), and water in the last year before cancer diagnosis were collected. For each food item, the reported frequency of consumption (daily, weekly, monthly, or yearly) was converted to frequency per day and was multiplied by the standard portion size (grams) using household measures to calculate grams per day. Using the food composition database developed for the Iranian population based on USDA food composition (27), Near-East food composition (28), and Bahrain food composition (29), macronutrients and micronutrients were calculated.

Regarding this analysis, we calculated the intake of 50 dietary fats based on our food composition table. According to previous studies (21, 22, 30) and the importance and commonplace of FAs in daily food, the gram per day of following FAs were considered in this study: (a) total fat; (b) cholesterol; (c) total SFAs, myristic acid pentadecanoic acid, palmitic acid, heptadecanoic acid, stearic acid; (d) total MUFAs, palmitoleic acid, oleic acid; (e) total PUFAs.

### Statistical analyses

The means and standard deviations (±SD) for continuous variables and the frequencies for categorical variables were calculated for descriptive statistics of baseline characteristics and intake of dietary factors of cases and controls.

The normality tested by comparing a histogram of the data to a normal probability curve. After examining the distribution of the data, all nutrient intakes were log-transformed to improve normality.

For the analysis of the association between dietary fat and different type of FFAs; SFAs, PUFAs, MUFAs, and cholesterol and CRC, we conducted unconditional multivariable logistic regression models to estimate the odds ratios (ORs) and 95% CIs. Before multivariate logistic regression, correlations between different exposures were checked and ORs were adjusted by gender (male/female), age (continuous), province, BMI (continuous), tobacco (smoking and water pipe) consumption (Never /Ever), aspirin use (Yes/No), opium use (no user / irregular users / regular users'), SES (Low/ Medium/ High), work-related physical activity (sedentary/Moderate/Heavy), processed meat intake including mortadella, hamburger and sausage (continuous, g/day), calcium (continuous, mg/day), fiber intake (continuous, g/day), and energy (continuous, kcal/day). Participants with missing data for physical activity (24.93 %) were coded as distinct categories during the analysis. Quartile were calculated based on the distribution of different type of fat intake between controls of study. Besides the main analysis, continuous analyses were also run across quartiles. Furthermore, analyses were stratified by age (under and over 50), BMI, gender, SES, physical activity, and vegetable intake for comparing quartile 4 to quartile1 of different types of fat intake. Nevertheless, according to significant interaction (P-heterogeneity < 0.05) analysis we only report age category results. We repeated the analyses for all CRC patients and then after stratification for sub-sites, including colon overall, proximal colon, distal colon, and rectum. All statistical analyses were carried out using Stata 14 (Stata Statistical Software: Release 14. College Station, TX: Stata Corp LLC). We considered *p* values <0.05 as statistically significant.

### Ethical considerations

The study was approved by the Ethics Committee of the National Institute for Medical Research Development (NIMAD) (Code: IR.NIMAD.REC.1394.027). All participants signed a written informed consent to participate in the study.

## Results

The current study included a total of 865 incident CRC cases, 434 cancers of the colon, 404 rectal cancer cases, 27 cases from unknown sub-site, and 3,206 controls; their socio-demographic characteristics, dietary factors intakes, and the distribution of selected CRC risk factors are shown in Table 1. One-third (*N* = 145) of the colon cancers were in the proximal colon, 42% (*N* = 185) in the distal colon, and 23% (*N* = 104) to the overlapping region between proximal and distal colon. In our study, the male to female ratio was 2, and around 76% of all participants were older than 50 years of age. Fars and Tehran provinces had the largest share of the study population. Significant differences between cases and controls were observed for tobacco use, family history, socioeconomic status, and opium use, but not for BMI and physical activity. CRC cases reported higher dietary energy intake (2,406 kcal/day) compared to controls (2,319 kcal/day). Higher levels of FAs and cholesterol were reported by cases than controls (Table 1).

**Table 1:** Selected baseline demographic and lifestyle characteristics of study participants by colorectal cancer status, IROPICAN study.

	**Controls**	**Cases**		
		Colorectal^*^	**Colon**	**Rectum**
**Overall**	3,206	865	434	404
**Province**, ***N*** **(%)**				
Tehran	816 (25.45%)	165 (19.08%)	101 (23.27%)	64 (15.84%)
Fars	943 (29.41%)	248 (28.67%)	93 (21.43%)	155 (38.37%)
Kerman	525 (16.38%)	100 (11.56%)	49 (11.29%)	51 (12.62%)
Golestan	373 (11.63%)	149 (17.23%)	89 (20.51%)	53 (13.12%)
Mazandaran	136 (4.24%)	59 (6.82%)	34 (7.83%)	25 (6.19%)
Kermanshah	251 (7.83%)	68 (7.86%)	31 (7.14%)	35 (8.66%)
Mashhad	162 (5.05%)	76 (8.79%)	37 (8.53%)	21 (5.20%)
**Gender**, ***N*** **(%)**				
Women	1,003 (31.28%)	368 (42.54%)	193 (44.47%)	169 (41.83%)
Men	2,203 (68.71%)	497 (57.46%)	241 (55.53%)	235 (58.17%)
**Age, years**, ***N*** **(%)**				
<30	21 (0.66%)	8 (0.92%)	3 (0.69%)	5 (1.24%)
≥30 & <40	227 (7.08%)	60 (6.94%)	32 (7.37%)	27 (6.68%)
≥40 & <50	503 (15.69%)	126 (14.57%)	64 (14.75%)	58 (14.36%)
≥50 & <60	993 (30.97%)	242 (27.98%)	112 (25.81%)	123 (30.45%)
≥60 & <70	1,020 (31.82%)	258 (29.83%)	137 (31.57%)	112 (27.72%)
≥70	442 (13.79%)	171 (19.77%)	86 (19.82%)	79 (19.55%)
**Socio-economic status (SES)**, ***N*** **(%)**				
Low	861 (26.86%)	337 (38.27%)	159 (36.64%)	161 (39.85%)
Moderate	1,078 (33.62%)	234 (27.05%)	118 (27.19%)	109 (26.98%)
High	1,267 (39.52%)	300 (34.68%)	157 (36.18%)	134 (33.17%)
**Tobacco consumption**, ***N*** **(%)**				
No	2,153 (67.16%)	629 (72.72%)	334 (76.96%)	274 (67.82%)
Yes	1,053 (32.84%)	236 (27.28%)	100 (23.04%)	130 (32.18%)
**Opium consumption**, ***N*** **(%)**				
No user	2,646 (82.53%)	731 (84.51%)	369 (85.02%)	340 (84.16%)
Regular user	432 (13.47%)	88 (10.17%)	40 (9.22%)	46 (11.39%)
Irregular user	128 (3.99%)	46 (5.32%)	25 (5.76%)	18 (4.46%)
**Work-related physical activity**, ***N*** **(%)**				
Sedentary	1,034 (32.27%)	287 (33.18%)	147 (33.87%)	132 (32.67%)
Moderate	701 (21.88%)	155 (17.92%)	78 (17.97%)	72 (17.82%)
Heavy	694(21.66%)	184 (21.27%)	87 (20.05%)	87 (21.53%)
Unknown	775 (24.19%)	239 (27.63%)	122 (28.11%)	113 (27.97%)
**Family history**, ***N*** **(%)**				
No	2,534 (79.04%)	653 (75.49%)	330 (76.04%)	304 (75.25%)
Yes	672 (20.96%)	212 (24.51%)	104 (23.96%)	100 (24.75%)
**Aspirin use**, ***N*** **(%)**				
No	2,469 (77%)	709 (81.97%)	358 (82.49%)	327 (80.94%)
Yes	737 (22.99 %)	156 (18.03%)	76 (17.51%)	77 (19.06%)
BMI^*^, kg/m ^**2**^ **, mean (±SD)**	26.59 (± 4.72)	26.93 (±4.99)	26.91 (±5.07)	26.83 (±4.85)
**Dietary intake, mean (±SD)**				
Total processed meat (g/day)	1.99 (± 0.12)	2.16 (±0.26)	2.47 (±0.43)	1.83 (±0.31)
Fiber (g/day)	24.72 (±11.25)	25.86 (±12.38)	25.28 (±12.16)	26.34 (±12.73)
Calcium (mg/day)	860.35 (±6.61)	908.28 (±14.69)	919.66 (±20.89)	880.24 (±21.16)
Dietary energy intake (kcal/day)	2,319.45 (±878.09)	2,405.61 (±1,076.00)	2,387.21 (±1,081.89)	2,393.21 (±1,066.35)
**Dietary intakes of Fatty acids, mean(±SD)**				
**Total fat (g/day)**	68.52 (±29.92)	77.39 (±39.70)	79.40 (±39.71)	74.19 (±39.29)
**Cholesterol (mg/day)**	247.21 (±154.15)	275.53 (±174.84)	285.77 (±181.61)	263.41 (±169.04)
Total SFAs^**^(g/day)	24.31 (±11.28)	27.72 (±14.89)	28.52 (±14.44)	26.32 (±15.10)
14:0 (Myristic acid) (g/day)	2.51 (±1.26)	2.82 (±1.56)	2.90 (±1.52)	2.66 (±1.56)
15:0 (Pentanoic acid) (g/day)	0.094 (±0.071)	0.09 (±0.07)	0.108 (±0.086)	0.088 (±0.067)
16:0 (Palmitic acid) (g/day)	12.79 (±5.95)	14.73 (±8.05)	15.12 (±7.77)	14.04 (±8.25)
17:0 (Heptanoic acid) (g/day)	0.078 (±0.052)	0.08 (± 0.06)	0.091 (±0.062)	0.078 (±0.056)
18:0 (Stearic acid) (g/day)	5.52 (±2.81)	6.37 (±3.75)	6.56 (±3.62)	6.046 (±3.84)
Total MUFAs^***^(g/day)	24.79 (±11.83)	28.38 (±15.83)	29.08 (±15.89)	27.27 (±15.62)
16:1n-7 cis (Palmitoleic acid) (g/day)	1.23 (±0.70)	1.50 (±0.99)	1.51 (±0.94)	1.45 (±1.05)
18:1n-9 cis (Oleic acid) (g/day)	22.95 (±11.01)	26.21 (±14.67)	26.88 (±14.76)	25.17 (±14.42)
Total PUFAs^****^(g/day)	12.27 (±5.66)	13.44 (±6.83)	13.87 (±7.19)	12.90 (±6.44)

* Includes 27 cancers with unknown sub-site.

BMI, body mass index;

** SFAs, saturated fatty acid;

*** MUFAs, monounsaturated fatty acids;

**** PUFAs, polyunsaturated fatty acids.

There was a statistically significant positive association between CRC and high intake (quartile 4) of dietary total fat (mean_Q4_ = 111 g/day, OR_Q4_ = 1.77, 95% CI = 1.32–2.38), cholesterol (mean_Q4_ = 437 mg/day, OR_Q4_ = 1.58, 95% CI = 1.22–2.05), and palmitoleic acid (mean_Q4_ = 2.24 g/day, OR_Q4_ = 2.16, 95% CI = 1.19–3.91), as well as an inverse association with high intake of dietary heptanoic acid (mean_Q1_: 0.147 g/day, OR_Q4_ = 0.33, 95% CI = 0.14, 0.79), low intake (quartile 2) of palmitic acid (mean_Q2_ = 10.45 g/day, OR_Q2_ = 0.53, 95% CI = 0.31–0.88) (Table 2).

**Table 2:** Dietary estimates of fatty acids and risk of colorectal cancer.

	**Q1**		**Q2**		**Q3**		**Q4**		OR (95% CI)^*^	***p*-trend**
**Fatty acids type**	**Mean**	**OR (95% CI)**	**Mean**	**OR (95% CI)**	**Mean**	**OR (95% CI)**	**Mean**	**OR (95% CI)**		
	**g/day**		**g/day**		**g/day**		**g/day**			
**Total fat**	37.4	1	56.49	0.95 (0.75–1.22)	72.26	0.91 (0.70–1.19)	110.74	**1.77 (1.32–2.38)**	**1.18 (1.07–1.30)**	**< 0.001**
**Cholesterol (mg/day)**	115	1	188.38	0.90 (0.71–1.14)	254.03	0.89 (0.69–1.14)	437.32	**1.58 (1.22–2.05)**	**1.15 (1.06–1.25)**	**0.001**
**Total SFAs**	12.6	1	19.92	0.77 (0.57–1.05)	25.87	0.98 (0.67–1.43)	40.08	1.59 (0.98–2.57)	1.14 (0.98–1.34)	0.081
14:0 (Myristic acid)	1.17	1	2.01	1.19 (0.76–1.87)	2.72	1.26 (0.66–2.38)	4.23	1.31 (0.58–2.95)	1.14 (0.89–1.47)	0.28
15:0 (Pentanoic acid)	0.028	1	0.065	0.76 (0.39–1.47)	0.107	0.99 (0.34–2.89)	0.179	1.52 (0.45–5.14)	1.24 (0.86–1.77)	0.234
16:0 (Palmitic acid)	6.67	1	10.45	**0.53 (0.31–0.88)**	13.56	0.83 (0.40–1.69)	21.19	1.01 (0.40–2.52)	0.94 (0.71–1.25)	0.695
17:0 (Heptanoic acid)	0.029	1	0.059	0.76 (0.47–1.24)	0.082	0.59 (0.30–1.14)	0.147	**0.33 (0.14–0.79)**	**0.74 (0.57–0.97)**	**0.034**
18:0 (Stearic acid)	2.68	1	4.39	1.28 (0.78–2.09)	5.82	0.56 (0.28-1.10)	9.47	0.91 (0.39–2.12)	0.94 (0.73–1.22)	0.695
**Total MUFAs**	12.78	1	19.86	1.26 (0.93–1.72)	26.18	0.90 (0.59–1.37)	41.75	0.96 (0.57–1.64)	0.98 (0.82–1.16)	0.836
16:1n-7 cis (Palmitoleic acid)	0.55	1	0.93	1.34 (0.94–1.91)	1.31	1.49 (0.92–2.42)	2.24	**2.16 (1.19–3.91)**	**1.22 (1.02–1.47)**	**0.027**
18:1n-9 cis (Oleic acid)	11.79	1	18.34	1.39 (0.93–2.07)	24.23	0.96 (0.54–1.71)	38.72	0.73 (0.35–1.54)	0.88(0.70–1.10)	0.271
**Total PUFAs**	6.5	1	9.87	0.93 (0.71–1.22)	12.84	0.95 (0.69–1.31)	20.16	1.25 (0.85–1.84)	1.06 (0.94–1.20)	0.301

* OR for the continuous analysis across quartiles.

Adjusted by province, age, SES, gender, BMI, tobacco use, opium use, aspirin use, physical activity, processed meat, fiber intake, calcium, and energy intake.

The bold values indicates the statistically significant results with *p* values < 0.05.

Additional analyses were performed on specific subsites of CRC cases. CRC were classified as colon, proximal or distal colon cancer, and rectum cancer. Based on stratified analyses by anatomical site, we identified a positive association between total fat and colon cancer [OR comparing highest to lowest quartile (Q4 vs. Q1) = 1.30, 95% CI = 1.14–1.48] as well as proximal [OR_Q4vs.Q1_ = 1.43, 95% CI; 1.15–1.77], and distal colon [OR_Q4vs.Q1_ = 1.25, 95% CI = 1.03–1.50].

Also, cholesterol intake was positively associated with colon [OR_Q4vs.Q1_ = 1.22, 95% CI = 1.09–1.36] and proximal colon cancer risk [OR_Q4vs.Q1_ = 1.25, 95% CI = 1.04–1.51].

**Figure 2:**
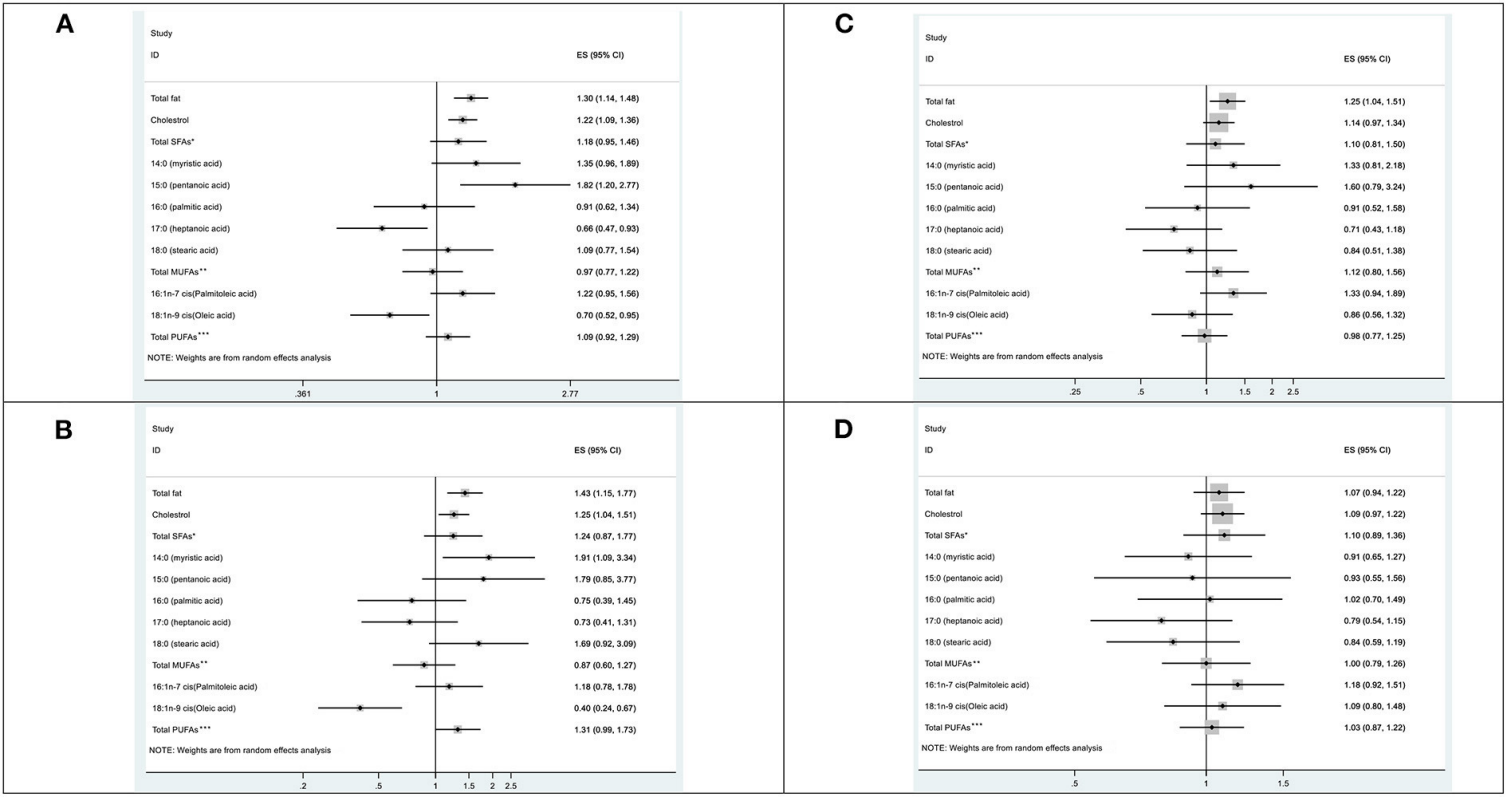
Forst plots according to the odds ratios, and 95% confidence intervals for colorectal cancer risk associated with different fat intake, by anatomical sub-site; **(A)** Colon cancer, **(B)** Proximal colon cancer, **(C)** Distal colon cancer, **(D)** Rectum cancer. *Saturated fatty acids; **Monounsaturated fatty acids; ***Polyunsaturated fatty acides.

Total SFAs intake was not associated with different sub-anatomical location of colon cancer, but we found a positive association for individual SFAs: pentanoic acid and colon cancer [OR_Q4vs.Q1_ = 1.81, 95% CI = 1.14–2.87], myristic acid [OR_Q4vs.Q1_ = 1.91, 95% CI = 1.09–3.34] and proximal colon cancer. Also, an inverse association was observed for heptanoic acid and colon cancer [OR_Q4vs.Q1_ = 0.66, 95% CI = 0.47–0.94]. Intake of oleic acid, which is one of the important MUFAs, was inversely associated with colon [OR_Q4vs.Q1_ = 0.70, 95% CI = 0.52–0.95] and proximal colon cancer risk [OR_Q4vs.Q1_ = 0.50, 95% CI = 0.30–0.83]. Conversely, no significant associations were observed for total MUFAs and PUFAs. Rectal cancer was not associated with any FA (Table 3 and Figure 2). A further analysis between different types of FAs and all sub-anatomical locations of CRC according to different quartiles indicated a positive association between stearic acid and proximal colon cancer [ORQ4 = 8.33; 95% CI = 1.02–67.65].

**Table 3:** Odds ratios and 95% confidence intervals of colorectal cancer and specific fatty acids intakes stratified by colorectal tumor location.

**Fatty acids type**	**Anatomical cancer site**			
	**Colon** OR (95% CI)^*^	**Proximal colon** OR (95% CI)^*^	**Distal colon** OR (95% CI)^*^	**Rectum** OR (95% CI)^*^
Total fat	**1.30 (1.14–1.48)**	**1.43 (1.15–1.77)**	**1.25 (1.03–1.50)**	1.07 (0.94–1.23)
Cholestrol	**1.22 (1.09–1.36)**	**1.25 (1.04–1.51)**	1.14 (0.97–1.35)	1.09 (0.97–1.22)
Total SFAs^*^	1.18 (0.95–1.46)	1.24 (0.87–1.77)	1.10 (0.81–1.50)	1.10 (0.89–1.37)
14:0 (Myristic acid)	1.35 (0.96–1.89)	**1.91 (1.09–3.34)**	1.33 (0.81–2.18)	0.91 (0.65–1.27)
15:0 (Pentanoic acid)	**1.81 (1.14–2.87)**	1.79 (0.85–3.77)	1.60 (0.79–3.23)	0.93 (0.55–1.55)
16:0 (Palmitic acid)	0.91 (0.62–1.34)	0.75 (0.39–1.46)	0.91 (0.52–1.57)	1.02 (0.70–1.50)
17:0 (Heptanoic acid)	**0.66 (0.47–0.94)**	0.73 (0.41–1.32)	0.71 (0.43–1.19)	0.79 (0.54–1.14)
18:0 (Stearic acid)	1.09 (0.77–1.54)	1.69 (0.92–3.08)	0.84 (0.51–1.38)	0.84 (0.59–1.19)
**Total MUFAs**	0.97 (0.77–1.22)	0.87 (0.59–1.26)	1.12 (0.80–1.56)	1.00 (0.79–1.26)
16:1n-7 cis (Palmitoleic acid)	1.22(0.96–1.57)	1.18 (0.78–1.77)	1.33 (0.94–1.90)	1.18 (0.92–1.50)
18:1n-9 cis (Oleic acid)	**0.70 (0.52–0.95)**	**0.50 (0.30–0.83)**	0.86 (0.56–1.32)	1.09 (0.80–1.48)
**Total PUFAs**	1.09 (0.92–1.29)	1.31 (0.99–1.72)	0.98 (0.77–1.25)	1.03 (0.87–1.22)

* OR for the continuous analysis across quartiles.

Adjusted by province, age, SES, gender, BMI, tobacco use, opium use, aspirin use, physical activity, processed meat, fiber intake, calcium, energy intake.

The bold values indicates the statistically significant results with *p* values <0.05.

According to the analyses stratified by age category, the statistically significant association persisted for total fat and CRC [OR_Q4vs.Q1_ = 1.30, 95% CI = 1.16–1.46, *p* = 0.006 for the interaction], as well as for colon [OR_Q4vs.Q1_ = 1.38, 95% CI = 1.18–1.61, *p* = 0.024 for the interaction] in subjects older than 50. Furthermore, a significant positive association was reported between cholesterol with CRC [OR_Q4vs.Q1_ = 1.22, 95% CI = 1.11–1.35, *p* = 0.019 for the interaction], colon [OR_Q4vs.Q1_ = 1.30, 95% CI = 1.14–1.48, *p* = 0.038 for the interaction]. There was no association between FAs intake and any of the cancer sub-sites among participants under 50 years old (Table 4).

**Table 4:** Odds ratios and 95% confidence intervals of colorectal cancer and specific fatty acids intakes stratified by age.

**Fatty acids type**	**Cancer site**	**Age**			
		**Number of cases**	**≤50** ***N* = 1,077** **OR (95% CI)**	**Number of cases**	**>50** ***N* = 2,994** **OR (95% CI)**
Total fat	**Colorectal**	206	0.89 (0.74–1.07)	659	**1.30 (1.16–1.46)**
	**Colon**	104	1.03 (0.80–1.32)	303	**1.38 (1.18–1.61)**
	Proximal colon	35	1.02 (0.67–1.55)	110	**1.62 (1.25–2.11)**
	Distal colon	43	1.00 (0.69–1.45)	142	**1.32 (1.05–1.65)**
	**Rectum**	97	0.79 (0.61–1.02)	307	**1.22 (1.04–1.44)**
Cholesterol	**Colorectal**	206	0.97 (0.81–1.16)	659	**1.22 (1.11–1.35)**
	**Colon**	104	0.99 (0.78–1.25)	303	**1.30 (1.14–1.48)**
	Proximal colon	35	0.97 (0.65–1.45)	110	**1.36 (1.10–1.69)**
	Distal colon	43	0.98 (0.68–1.41)	142	**1.21 (1.00–1.46)**
	**Rectum**	97	0.98 (0.77–1.25)	307	**1.14 (1.00–1.30)**
Total SFAs	**Colorectal**	206	1.13 (0.82–1.56)	659	1.16 (0.97–1.39)
	**Colon**	104	1.29 (0.83–2.01)	303	1.15 (0.90–1.48)
	Proximal colon	35	1.52 (0.71–3.23)	110	1.22 (0.81–1.84)
	Distal colon	43	1.42 (0.74–2.72)	142	1.01 (0.71–1.44)
	**Rectum**	97	0.96 (0.62–1.48)	307	1.19 (0.93–1.53)
Total MUFAs	**Colorectal**	206	0.79 (0.55–1.14)	659	1.04 (0.86–1.27)
	**Colon**	104	0.72 (0.45–1.17)	303	1.06 (0.81–1.38)
	Proximal colon	35	0.53 (0.23–1.21)	110	0.98 (0.63–1.51)
	Distal colon	43	0.73 (0.36–1.48)	142	1.28 (0.88–1.88)
	**Rectum**	97	0.96 (0.58–1.56)	307	1.01 (0.78–1.32)
Total PUFAs	**Colorectal**	206	1.01 (0.78–1.31)	659	1.07 (0.92–1.23)
	**Colon**	104	1.15 (0.82–1.62)	303	1.05 (0.86–1.27)
	Proximal colon	35	1.35 (0.76–2.38)	110	1.31 (0.95–1.79)
	Distal colon	43	1.17 (0.70–1.94)	142	0.90 (0.68–1.18)
	**Rectum**	97	0.84 (0.59–1.20)	307	1.09 (0.90–1.33)

The bold values indicates the statistically significant results with *p* values <0.05.

Adjusted by province, age, SES, gender, BMI, tobacco use, opium use, aspirin use, physical activity, processed meat, fiber intake, calcium, energy intake.

## Discussion

We found a positive relationship between total fat, cholesterol, myristic acid, pentanoic acid, and a high intake of palmitoleic acid, with CRC, with a stronger effect on colon and proximal colon cancer. The associations were stronger in subjects older than 50 and absent in younger ones. A high intake of heptanoic acid, Oleic acid, and a low intake of palmitic acid showed inverse associations with CRC and colon cancer. We couldn't find any relation between rectal cancer and different kinds of FAs. Total PUFAs did not appear to have a significant effect on CRC in our study.

High dietary fat intake resulted in an increase in CRC risk of about 60%. The effect appeared to be exerted on the colon rather than the rectum, especially on the proximal section rather than the distal. Studies also have shown that FAs have different effects depending on the anatomical region of the colon (proximal and distal) and the source of the fat (14, 20, 21, 30–32). For example, in our study, plant-sourced fats such as heptanoic acid and oleic acid, or animal fats such as palmitic acid, stearic acid, and myristic acid obtained from meat and dairy products, show different effect on sub-site of colon and rectum. Indeed, a large body of literature described the enhanced risk of colon cancer with high fat intake (16, 33, 34), despite several studies finding no association between FAs and CRC overall (35). A recent meta-analysis found weak evidence of a correlation between FAs and CRC in Chinese men (36). According to a world cancer research fund international (WCRF) report published in 2018, evidence of the association between fats and different fatty acid types is limited and requires further investigation (22).

A number of mechanisms contributed to the effect of FAs on CRC risk, such as (1) pro-inflammatory effects that may be triggered by interaction between dietary fat and gut microbiota, which play a role in the metabolism of bile acids (BA) (37, 38). In fact, BA supports the concept of CRC. Evidence of selective uptake of PUFAs from CRC cells have been provided (39). (2) Oils and fats as the highest sources of polycyclic aromatic hydrocarbons (PAH) (40) can damage DNA in several target tissues when consumed through saturated fat (41). (3) A higher intake of saturated fat has been associated with increased oxidative damage and lipid peroxidation based on the *in-vivo* and *in-vitro* studies (42, 43). (4) Lipids have also been shown to affect cell membrane structure and function, signaling pathways, and gene expression (44). (5) Moreover, obesity is a major risk factor for CRC, with a preponderant role of visceral adiposity (45). A double-blind trial reported subjects overfed with highly SFAs foods underwent more visceral adiposity accumulation than subjects overfed with foods rich in PUFAs showing a distinct effect on fat accumulation in humans, which can in turn be differently related to CRC (46).

Our results showed IROPICAN study population reported a higher daily intake of total fat (70 grams/day, 27% of kcal energy), cholesterol (253 mg/day), and saturated fat (25 grams/day, 10% of kcal energy) than recommendations, coupled with a lower intake of total PUFAs (5% of total kcal energy), reflecting previous studies regarding the amount of dietary fat from different sources in Iranian populations (47, 48). The benefit of unsaturated FA, PUFAs, and MUFAs derived from an overall positive metabolic effect, with inhibition of inflammation and balance of microbiota composition (49). Total MUFAs and total PUFAs do not appear to be associated with CRC in our study. Fish and oily fish are the main sources of ω3 PUFAs (50), while ω6 PUFAs are obtained from vegetable oil, nuts, and egg (51). In terms of fish and seafood consumption, there is a big difference between Iran and other countries, and most PUFAs in the Iranian diet are supplied from other sources such as liquid vegetable oils (Sunflower, maize oil) (52, 53). For human health, it is essential to have a balanced ratio between the two types of PUFAs (54, 55). In this study, we did not investigate the effect of different types of PUFAs on CRC, as we plan to address this topic in a separate paper.

Over time, the type of oil available on the Iranian market has changed. According to a WHO report published in 2018, Iranian households consume a great deal of cooking oil made from partially hydrogenated vegetable oils, a major source of trans fatty acids (TFAs) (56). In recent years, many activities have been conducted with the goal of increasing awareness of solid/semisolid hydrogenated oils and reducing their consumption in Iran (57, 58). People who are 50 or older seem to use more of this type of fat than people who are younger. TFAs could influence cholesterol balance and the effect exerted by the amount of total fat intake (59). This can be seen in our results, where total fat increases the risk of CRC according to the proportion of TFAs intake. Nevertheless, this suggestion requires further studies.

To our knowledge, this is the first study to investigate the magnitude of different types of FAs on CRC in a large Iranian population from different provinces and in the EMRO region. Our data are characterized by high quality because (i) information were collected by trained interviewers, (ii) same standardized validated FFQs and questioning tools were used in the different centers, and (iii) all cases were provided with pathological confirmation and allowed us analyses by subsites.

Two potential limitations of our study include: (i) selection bias, because the controls were not chosen on a population-based approach in the primary study (IROPICAN) but were rather taken among the healthy visitors who did not have CRC or other diseases. However, our validation study showed that due to appropriate using healthy visitors instead of disease controls such bias is minimal (60); (ii) reporting and recall bias, especially regarding the FFQ, because of the dependence on memory and possibly case-control status. However, it is likely that this bias might have operated in a similar way in cases and controls, resulting in non-differential misclassification and underestimation of the associations. Furthermore, diet might have changed among cases because of disease development. To allay this concern and risk of reverse causation bias, we collected dietary information one year before the data of cancer diagnosis among cases. Also, food composition tables may have some limitations, and matching is not always straightforward, so that the results may be impaired by residual confounding. Anyway, the different results between colon and rectal cancer argue against a strong role of selection or information bias. Finally, we could not report different group of FAs based on specific source such as animal, vegetables, seeds, so on. Future studies should explore the relationship between different type of FAs and CRC by considering the source intake. Also, comparing dietary and plasma levels of FAs can be interesting.

### Conclusion

In this large CRC case-control study, total fat, cholesterol, and higher intake of myristic acid, palmitic acid, palmitoleic acid from animal sources were associated with increased risk of CRC, and some FAs from plant sources such as heptanoic acid or oleic acid- the main FA in olive oil- decreased particularly colon cancer risk, after accounting for major adjustments. Moreover, subgroup analyses by age revealed that participants older than 50 years had a higher risk of CRC due to consumption of a high FAs diet. This may be due to the cohort effect and changes on the amount, and type of FAs intake over time. Our study improves general knowledge on CRC epidemiology and offers important insights on CRC in Iranian population. These data may be useful for the identification of high-risk individuals and public awareness to promote prevention of CRC in Iran and other LMICs that facing the increasing pattern in the incidence of CRC. In line with international evidence, the promotion of a decrease in fat consumption, especially FAs from industrial and animal sources, may decrease the risk of CRC among the Iranian population. Along these lines, a number of restrictions have been imposed in Iran since 1025 on oil products, including trans and saturated fat, aimed at controlling and preventing non-communicable diseases, including CRC (61).

## Data availability statement

The raw data supporting the conclusions of this article will be made available by the authors, without undue reservation.

## Ethics statement

The study was approved by the Ethics Committee of the National Institute for Medical Research Development (NIMAD) (Code: IR.NIMAD.REC.1394.027). Written informed consent was obtained from the individual(s) for the publication of any potentially identifiable images or data included in this article.

## Author contributions

MSS, KZ, and PB designed research. MSS, KZ, RA-N, RS-F, HR, and MH conducted research. MSS, GC, KZ, and PB analyzed data, performed statistical analysis, and wrote the paper. FK, AE, MG, VC, IH, and EP contributed in the editing of the preliminary results and draft. KZ had primary responsibility for final content. All authors read and approved the final manuscript.

## Funding

This study was funded by National Institute of Medical Research Development (NIMAD) (Code: IR.NIMAD.REC.1394.027) and particularly supported by Investigator Grant No. 24706 of Fondazione AIRC. Italy.

## Conflict of interest

The authors declare that the research was conducted in the absence of any commercial or financial relationships that could be construed as a potential conflict of interest.

## Publisher's note

All claims expressed in this article are solely those of the authors and do not necessarily represent those of their affiliated organizations, or those of the publisher, the editors and the reviewers. Any product that may be evaluated in this article, or claim that may be made by its manufacturer, is not guaranteed or endorsed by the publisher.

## Author disclaimer

Where authors are identified as personnel of the International Agency for Research on Cancer / World Health Organization, the authors alone are responsible for the views expressed in this article and they do not necessarily represent the decisions, policy or views of the International Agency for Research on Cancer / World Health Organization.

## Abbreviations

ASR: age-standardized incidence rate
BMI: body mass index
CI: confidence interval
CRC: colorectal cancer
EMRO: Eastern Mediterranean Region
FAs: fatty acids
FFAs: free fatty acids
FFQ: Food Frequency Questionnaire
LMIC: low and middle-income countries
MUFAs: monounsaturated fatty acids
OR: odds ratio
PUFAs: polyunsaturated fatty acids
SFAs: saturated fatty acid
TFAs: trans fatty acids
WCRF: World Cancer Research Fund.
